# Standardizing Recreational Cannabis Excise Tax Rates in the United States: New Retail Price-Based Measurements by Product Category

**DOI:** 10.3390/ijerph23010114

**Published:** 2026-01-16

**Authors:** Bing Han, Michael Cooper, Ce Shang, Yuyan Shi

**Affiliations:** 1 Herbert Wertheim School of Public Health and Human Longevity Science, University of California San Diego, 9500 Gilman Drive, La Jolla, CA 92093, USA; bihan@health.ucsd.edu (B.H.); m3cooper@health.ucsd.edu (M.C.); 2 Center for Tobacco Research, The Ohio State University Wexner Medical Center, Columbus, OH 43214, USA; ce.shang@osumc.edu; 3 Division of Medical Oncology, Department of Internal Medicine, The Ohio State University Wexner Medical Center, Columbus, OH 43214, USA

**Keywords:** recreational cannabis, cannabis legalization, price, excise tax, tax incidence, instrumental variable

## Abstract

**Highlights:**

**Public health relevance—How does this work relate to a public health issue?**

- Cannabis excise taxes have potential to influence cannabis demand and cannabis-related health outcomes.

**Public health significance—Why is this work of significance to public health?**

- Standardizing category-specific cannabis excise taxes across states may strengthen cross-state comparisons and improve evaluations of how cannabis taxes and prices influence public health outcomes.

**Public health implications—What are the key implications or messages for practitioners, policy makers and/or researchers in public health?**

- Substantial heterogeneities exist in cannabis excise tax structures, standardized taxes, and tax incidences across states.
- Category-specific cannabis excise taxes strongly predicted cannabis retail prices, supporting their use as an instrumental variable candidate.

**Abstract:**

Background: Cannabis excise tax structures vary widely across the states in the United States. Standardizing taxes may improve cross-state comparisons and strengthen evaluations of how taxes and prices influence public health outcomes. This study developed category-specific standardized tax metrics for flower, vaping, and edible products by incorporating price and tax structure variations using retail scanner data. Methods: We analyzed cannabis retail scanner data from dispensary point-of-sale systems for flower, vaping, and edible products in 12 states with legal recreational markets from Q1 2020 to Q4 2024. Using retail prices and excise tax policies, we converted taxes in different forms across the supply chain into standardized measures and estimated tax incidence (ratio of standardized taxes to retail prices) for each category. We also evaluated the association between standardized taxes and retail prices. Results: Mean standardized excise taxes were USD 32.58/ounce for flower, USD 180.21/ounce for vaping, and USD 0.024/milligram THC for edible products. Corresponding tax incidences were 13.03%, 13.59%, and 13.09%. Standardized taxes and tax incidences varied considerably across states. Category-specific standardized taxes strongly predicted retail prices, supporting their use as an instrumental variable candidate. Conclusions: Category-specific standardized measures of cannabis excise taxes derived from retail scanner data may support cross-state comparisons and pricing policy evaluation.

## 1. Introduction

Despite the continued federal prohibition of cannabis use and sales, an increasing number of states in the United States (U.S.) have legalized and commercialized cannabis. As of October 2025, 24 states and Washington, D.C., have legalized possession of small amounts of cannabis among adults, and 22 of these states have established legal marketplaces for adult-use sales. While recreational cannabis legalization and commercialization reflect evolving public attitudes and generate substantial economic activity, they also raise important public health concerns. A growing body of evidence links recreational cannabis legalization and commercialization to higher rates of cannabis use, cannabis use disorder, and cannabis-related healthcare encounters [1,2,3]. These trends underscore the need for regulatory measures that balance market expansion with the protection of public health.

Taxation has long been recognized as one of the most effective tools for reducing tobacco and alcohol use and their associated harms [4,5]. Compared with other regulatory approaches that require intensive enforcement, tax policies can be administered through existing fiscal infrastructures, making them relatively cost-effective. Moreover, tax revenues can be directed toward prevention, treatment, and education programs, creating a sustainable mechanism to support public health priorities [6].

All U.S. states with legal recreational cannabis markets impose statewide excise taxes, but tax structures vary widely in their bases, rates, and points of collection. Tax bases include (1) price-based taxes (ad valorem tax), levied as a percentage of prices; (2) delta-9-tetrahydrocannabinol (THC)-based taxes (specific tax), calculated as THC content (typically measured in milligram THC) multiplied by a tax rate; and (3) weight-based taxes (specific tax), calculated as product weight multiplied by a tax rate. Price-based taxes are the most common. Some states employ mixed systems that combine multiple bases. For instance, Illinois combined price-based and THC-based taxes: retail sales were taxed at 10% for products with THC concentrations below 35%, 25% for products with THC concentrations above 35%, and 20% for infused products (edibles). Tax rates within a tax base differ substantially. For example, price-based tax rates in the retail stage range from 3% to 37% of retail prices [7]. Excise taxes are also imposed at different points along the supply chain, including cultivation, wholesale, and retail stages. Evidence from tobacco control suggested that cultivation-stage taxes can be particularly effective in reducing tax evasion and improving compliance [8]. Wholesale taxes are generally passed downstream, ultimately influencing retail prices, while retail-stage taxes directly increase the prices consumers pay and can therefore have the most immediate impact on demand [9]. The absence of a unified tax model complicates cross-state comparisons of cannabis market activity and tax burdens, as well as evaluations of how tax policies shape revenue generation and public health outcomes.

Park et al. 2024 made the first attempt to standardize cannabis excise taxes on flower products across U.S. states [10]. Because tax calculations require price data from different stages of the supply chain, inconsistent data availability across states posed a major challenge. To address this, Park et al. integrated multiple data sources [10]. In a few states where governments published average prices at any stage of the supply chain, those publicly available data were used. For most states, however, such information was unavailable, and wholesale flower prices were obtained from a commercial data provider. To fill additional missing price observations in the cultivation and retail stages, Park et al. 2024 applied empirically derived assumptions: wholesale prices were converted to retail prices using a 97% markup rate based on observations from states with concurrent wholesale and retail price data, and cultivation prices were treated as equivalent to wholesale prices based on observations from states with concurrent cultivation and wholesale price data [10].

While Park et al. 2024 established an important framework for standardizing cannabis excise taxes across states, their approach had limitations [10]. First, their tax standardization focused exclusively on flower products. In recent years, however, non-flower products such as vaping and edible products have captured a substantial share of the U.S. legal market, accounting for 24% and 22% of total sales in 2023, respectively [11]. These products differ markedly from flower in tax structures, pricing, and potency, all of which influence the calculation of tax rates. To inform policy and research effectively, tax standardization should therefore be extended to specific product categories. Second, although nearly all states levy price-based taxes at the retail stage and a few at the cultivation or wholesale stage, only a quarter of states in Park et al. 2024 [10] provided government-reported retail price data. For the remaining states, retail prices were imputed from wholesale prices using a uniform markup rate. Such estimates are likely imprecise. Third, while their tax measures expressed in the form of USD per ounce are appropriate for flower and vaping products, they may not be appropriate for edibles, where product weight has little public health relevance and THC content is the primary determinant of health risk.

To extend the research by Park et al. 2024 [10] and address the identified gaps, the present study aimed to develop category-specific standardized excise tax metrics for flower, vaping, and edible products across U.S. states. This study makes several contributions. First, we incorporated price and tax structure variations across product categories to generate category-specific tax measures. This broader scope allows for more accurate and nuanced comparisons of cannabis excise taxes in increasingly diverse and complex marketplaces. Second, instead of imputing retail prices from wholesale data, we used retail prices collected directly from point-of-sale systems in cannabis dispensaries, offering empirically observed transaction data that better capture real market conditions and state-level variations. Third, this study is the first to standardize cannabis taxes on edibles in the form of USD per milligram THC, which is more directly relevant to public health outcomes. While most states currently levy price-based taxes, estimating excise taxes on a potency basis is important for informing policymakers who may consider alternative tax structures and providing a foundation for future research on potency-based taxation [12]. Finally, price is an endogenous variable in economics research because price is jointly determined by demand and supply. Excise taxes have been commonly employed as instrumental variables for prices in tobacco studies and recently applied in cannabis research [13,14,15]. We therefore evaluated the validity of the standardized tax measures as an instrument by testing whether these tax measures significantly impacted retail prices. This analysis may inform future cannabis policy research on the empirical validity of applying tax measures within causal inference models.

## 2. Materials and Methods

### 2.1. Cannabis Retail Price: Data Source

Point-of-sale cannabis retail scanner data were obtained from BDSA (Boulder, CO, USA), a market research firm specializing in U.S. cannabis industry analytics. BDSA compiles its proprietary dataset through the Retail Sales Tracking platform, which captures real-time retail scanner data directly from the point-of-sale systems of participating dispensaries. In each tracked state, approximately 40–50% of licensed dispensaries contribute to this system. The sample includes multi-store chains, large single-site dispensaries, and smaller independent outlets located in urban, suburban, and rural areas, thereby representing the broader state-level market. All participating dispensaries were state-licensed and actively operating at the time of data collection. To estimate statewide market activity, BDSA applies proprietary algorithms that project sales from the sampled dispensaries and calibrates the projections against total sales data published by state governments. BDSA has validated this projection methodology through internal tests using random subsamples in a state with complete dispensary coverage. Previous studies have used data from other cannabis market research firms that employed comparable projection strategies [16].

Although every state with legalized recreational cannabis markets actively tracks sales through Seed-to-Sale Tracking systems, these systems are managed independently by each state, with minimal data sharing across jurisdictions. Moreover, the data collected are rarely made publicly available. In the absence of complete direct sales data from all dispensaries, BDSA’s projected sales data currently provide the most comprehensive estimates of cannabis market activity in multiple states. Between Q1 2020 and Q4 2024, BDSA tracked retail scanner data in 12 of the 22 U.S. states with legal recreational markets: Arizona, California, Colorado, Illinois, Maryland, Massachusetts, Michigan, Missouri, Nevada, New Jersey, Ohio, and Oregon. These 12 states were included in the present study. The raw dataset contained 3,914,628 product–state–year-quarter observations, each recording tax-exclusive, post-discount total sales (USD), unit volume sold (e.g., packs, grams), and product characteristics for a given product in a state-year-quarter. THC content information was only available for edibles in the BDSA data. Products sold in different package sizes (e.g., 1 g vs. 3.5 g) were treated as separate observations.

Before statistical analysis, we applied several exclusions: (1) sales through medical channels (n = 1,169,578), except in California where medical and recreational sales were not distinguished in the BDSA data and were applied with the same excise tax structure; (2) non-edible products missing weight or package size information (n = 110,469); and (3) edible products missing THC content information (n = 40,713). After these exclusions, the dataset was reduced to 2,646,318 observations. We grouped these observations into four broad product categories: (1) flower (loose flower and pre-rolls), (2) vaping (extracts, cartridges, and disposables), (3) edibles (candies, beverages, culinary items, chocolates, baked goods, pills, and other edibles), and (4) other products (topicals, sublinguals, accessories, etc.). All four categories were included in the market share analysis.

For the remaining analysis involving retail prices, we focused on flower, vaping, and edibles, excluding other products (n = 213,860). Following standard practice [17,18], we further excluded price outliers within each category, defined as observations with prices below the 1st percentile or above the 99th percentile. These outliers likely reflected free giveaways or reporting errors. This step accounted for an additional 48,691 exclusions. The final analytic sample for retail price analysis consisted of 2,383,767 product–state–year-quarter observations, including 1,222,236 flower, 1,001,208 vaping, and 160,323 edible products.

### 2.2. Cannabis Tax Policies: Data Source

We compiled state-level cannabis excise tax policies from official government websites, and documented effective dates, subsequent updates, and the tax bases and rates applied at each stage of the supply chain (Table S1). Consistent with Park et al. 2024, we did not include general sales taxes in tax standardization, as they are generally applied to all goods and services and not considered to be a tool for public health protection [10]. Following previous conventions in the field [10,19], in states where cannabis was exempt from general state-level sales taxes (e.g., Colorado and Maryland), we adjusted the effective tax rate used for the standardization of retail-level price-based taxes by subtracting general state sales tax rate from retail-level price-based tax rate (Technical Note S1). The adjusted effective tax rate reflected the actual tax burden for cannabis products at the retail level. The same adjustment has been adopted by the National Institute on Alcohol Abuse and Alcoholism to publish effective tax rates for alcohol products, for which general state sales taxes are usually exempt. Market authorization did not always align with the implementation of tax policies. For instance, Oregon adopted its excise tax structure three months after authorizing the recreational cannabis marketplace. We therefore used the effective date of tax policy implementation as the starting point for tax standardization.

Substantial variation in tax structures (tax base, tax rate, and point of collection) was observed across the 12 states analyzed in this study (Table S1). With respect to tax base (price-, THC-, or weight-based), eight states relied exclusively on price-based taxes, and one state used only weight-based taxes. The remaining three states adopted mixed systems that combined two of these tax bases: two states employed a combination of price-based and weight-based taxes, and one state applied both price-based and potency-based taxes. Tax rate varied. For instance, it ranged between 6% and 25% at the retail stage. Variation also existed in the point of collection: one state levied taxes solely at the cultivation stage, seven states imposed excise taxes only at the retail stage, and three states applied taxes at multiple points along the supply chain.

Cannabis tax structures further varied by product category (Table S1). Flower products were commonly taxed from the cultivation to retail stages, whereas manufactured products such as vaping and edibles were typically taxed at the wholesale and retail stages, after processing and packaging. These distinctions likely reflected the complexity of value-added production and differences in product form.

### 2.3. Tax Standardization Procedure

We adapted the general procedure developed by Park et al. 2024 [10], introducing modifications to account for category-specific tax structures and the use of retail price data. Full details of the original procedure are available in Park et al. 2024 [10]. We only highlight our modifications below.

Because the vast majority of states adopted price-based taxes, estimating prices along the supply chain is essential for tax standardization. Unlike Park et al. 2024, which primarily relied on wholesale price estimates and extrapolated them to cultivation and retail prices [10], we used the BDSA retail scanner data to estimate retail prices and then extrapolated them to cultivation and wholesale stages. This approach was chosen for three reasons: (1) the wholesale price data source used by Park et al. 2024 [10] was limited to flower products and did not include other product categories; (2) alternative data sources for retail prices, such as publicly available government reports, were generally limited to flower products, not consistently available across states, and did not report prices in a standardized format; and (3) precise estimates of retail prices are more critical than cultivation or wholesale prices, since price-based taxes at the retail stage are far more common than those levied earlier in the supply chain.

Using the BDSA retail scanner data, we constructed the following measures of average tax-exclusive retail prices for each state in each year-quarter: (1) USD per ounce for flower products, calculated as total sales (USD) divided by total volume (ounce); (2) USD per ounce for vaping products, calculated as total sales (USD) divided by total volume (ounce); and (3) USD per milligram THC for edibles, calculated as total sales (USD) divided by the total THC content (milligram THC).

To extrapolate retail prices to cultivation and wholesale stages, we followed the procedure established by Park et al. 2024 [10]. Specifically, Park et al. 2024 [10] applied a 97% markup rate to convert wholesale to retail prices, based on data from states reporting both wholesale and retail prices. They also assumed cultivation prices were equivalent to wholesale prices, based on data from states where both were available. In the present study, similarly, conversion from retail to wholesale prices was performed using Equation (1), and conversion from wholesale to cultivation prices was performed using Equation (2)

*Wholesale price_sq_ = Retail price_sq_/(*1 *+ markup rate)* (1)

*Cultivation price_sq_ = Wholesale price_sq_* (2)

where *s* denotes state, and *q* denotes year-quarter. *Markup rate* was assumed to be 97%.

In our study sample, THC-based taxes were implemented only in Illinois at the retail stage. Illinois adopted a hybrid model that combined price-based and THC-based taxes: retail sales were taxed at 10% for products with THC concentrations below 35%, 25% for products with THC concentrations above 35%, and 20% for infused products (edibles). Because the BDSA data did not report THC content or concentration for flower and vaping products, we followed prior research and assumed an average THC concentration of 20% for flower products, applying a 10% tax rate to retail prices [10,17,20]. For vaping products, where THC concentrations have been estimated to average around 52% and can reach as high as 90% [21,22], we applied the 25% tax rate. For edibles in Illinois, we applied the 20% tax rate to retail prices and used the actual THC content reported in the BDSA data to convert taxes into USD per milligram of THC.

Effective excise taxes in each stage of the supply chain were calculated using Equation (3) for flower and vaping products and Equation (4) for edibles.

*Excise tax per oz_stage,sq_ = Weight-based tax per oz_stage,sq_ + Price-based tax rate_stage,sq_ × Price per oz_stage,sq_ (For Illinois: THC-tiered price-based tax rate_stage,sq_ × Price per oz_stage,sq_)* (3)

*Excise tax per mg THC_stage,sq_ = THC-based tax rate per mg THC_stage,sq_ + Price-based tax rate_stage,sq_ × Retail price per mg THC_stage,sq_ (For Illinois only: THC-tiered Price-based tax rate_stage,sq_ × Retail price per mg THC_stage,sq_)* (4)

where *stage* denotes stage in the supply chain (cultivation, wholesale, or retail), *s* denotes state, and *q* denotes year-quarter.

Finally, we used Equation (5) for flower and vaping products and Equation (6) for edibles to sum up the total excise taxes imposed with price-, THC-, and weight-bases in the entire supply chain (cultivation, wholesale, and retail stages).

*Standardized excise tax per oz_sq_ = Excise tax per oz_cultivation, sq_ + Excise tax per oz_wholesale, sq_* *+ Excise tax per oz_retail, sq_* (5)

*Standardized excise tax per mg THC_sq_ = Excise tax per mg THC_cultivation, sq_* *+ Excise tax per mg THC_wholesale, sq_ + Excise tax per mg THC_retail, sq_* (6)

where *s* denotes state, and *q* denotes year-quarter.

### 2.4. Statistical Analysis

All analyses were conducted at the state–year-quarter level by product category from Q1 2020 through Q4 2024. We first plotted trends in market share, retail prices, and standardized excise taxes, and mapped standardized excise taxes across states. To evaluate the persistence of time patterns in retail prices and standardized excise taxes, we tested whether these trends declined over time by regressing prices or taxes on a linear time trend. We also calculated tax incidence, defined as the ratio of standardized excise taxes to retail prices. Tax incidence is an indicator of consumer tax burden and tax policy strength and widely adopted in substance tax literature [23]. To assess how tax incidence was influenced by tax structure, we regressed tax incidence on both the tax magnitude and tax base indicators using simple linear regressions, controlling for year–quarter fixed effects.

An important application of standardized excise taxes is their use as instrumental variables for retail prices in analyses that estimate the impact of prices on demand [13]. Regressing retail prices on demand creates an endogeneity problem because the observed prices reflect both supply and demand conditions rather than an independent predictor. This endogeneity biases the estimated relationship between prices and demand and prevents a causal interpretation of the price coefficient. Excise taxes can serve as valid instruments because they directly affect the cost of a product and therefore shift retail prices, but they do not directly influence consumer demand except through those price changes. This provides exogenous variation in prices that can be used to identify the causal effect of price on demand. To assess whether our tax measures function as strong instruments, we estimated the following first-stage regression within a two-stage least squares framework.

(7) $$\mathrm{Retail}{\mathrm{price}}_{\mathrm{sq}}=\alpha \;+\;\beta \;\mathrm{Standardized excise}\;{\mathrm{tax}}_{\mathrm{sq}}\;+\;ɣZ_{\mathrm{sq}}\;+\;\delta_{s}+\;\mu_{q}+\;\nu_{sq}$$

where *s* denotes state and *q* denotes year-quarter. Z*_sq_* represents a vector of sociodemographic covariates (e.g., share of individuals aged 21 and older, racial/ethnic composition, unemployment rates, and poverty rates). $\delta_{s}$ and $\mu_{q}$ are state and year-quarter fixed effects, respectively. $\nu_{sq}$ is the error term. Standard errors are clustered at state level. The coefficient β captures the association between standardized excise taxes and retail prices. A common rule of thumb for a strong instrument is that the *F*-statistic for the instrumental variable (standardized excise taxes in this study) should exceed 10 [24].

A key concern regarding the use of price-based excise taxes as instruments for prices is that price-based taxes are correlated with prices. Following the approach of Park et al. 2024 [10], we also constructed alternative tax measures that were not affected by temporal fluctuations in prices. Specifically, we generated two category-specific measures: (1) taxes computed using overall average prices across the entire study period, and (2) taxes computed using prices observed in each state’s first available year, calculated by averaging data from the quarters available within that year.

To compare the performance of our category-specific standardized tax measures with the flower-specific tax measures developed by Park et al. 2024 [10], we reported descriptive statistics and tax-price regression results in the overlapping study period and state sample for both measures.

All the statistical analyses were conducted with Stata software (version 18).

## 3. Results

### 3.1. Cannabis Market Share and Retail Prices

Figure S1 shows trends in market share by product category. Flower products consistently accounted for the largest share, fluctuating around 50%. Vaping products increased steadily from 21.36% in 2020 to 26.31% in 2024, while edibles maintained a relatively stable share of about 14%.

**Figure 1:**
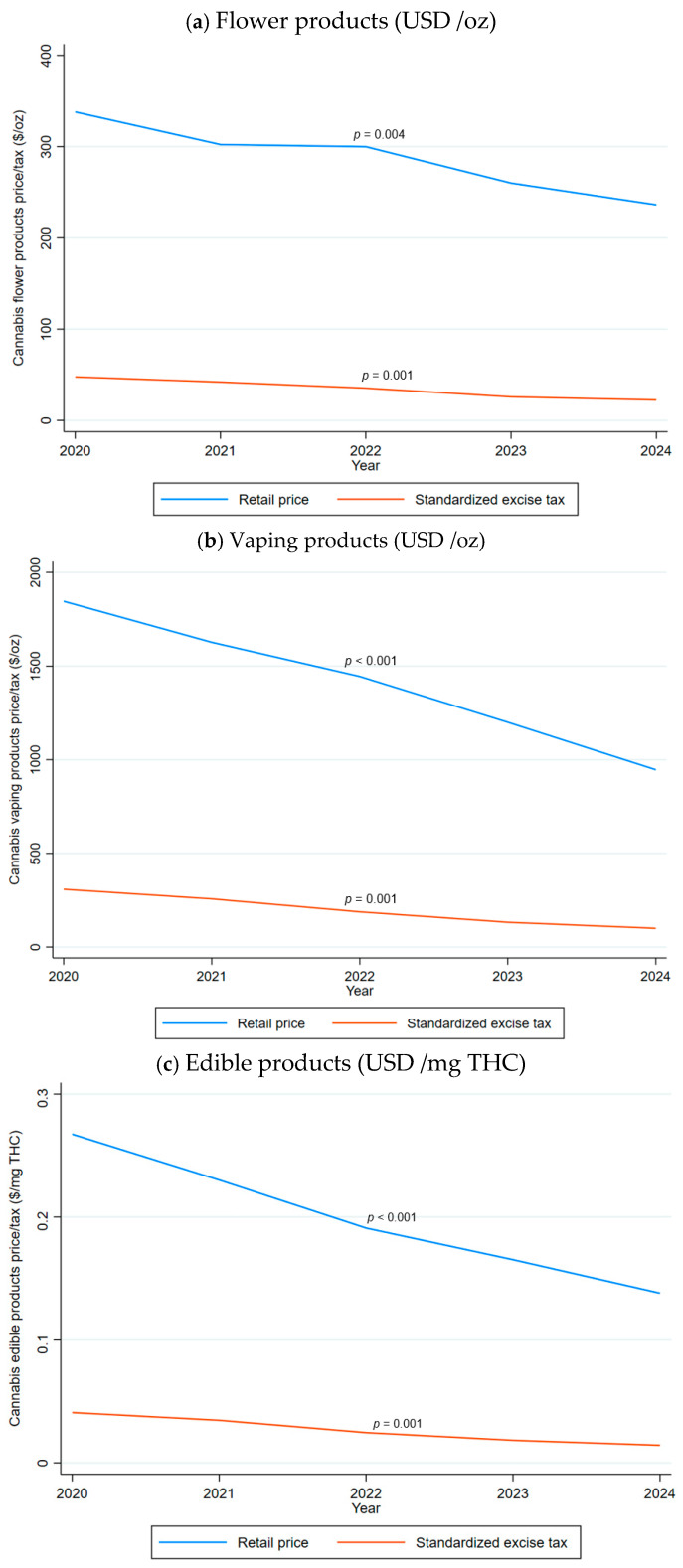
Trends in Cannabis Retail Prices and Standardized Excise Taxes by Product Category, 2020–2024 (inflation adjusted to 2024 dollars). Notes: *p*-values were calculated in a regression with a linear time trend as the only predictor. (**a**): flower products, (**b**): vaping products, (**c**): edible products.

Figure 1 displays trends in retail prices (inflation-adjusted to 2024 dollars) by product category. All three categories experienced substantial declines between 2020 and 2024 (all *Ps* for linear time trends < 0.01): flower prices decreased by 30.10%, vaping prices by 48.72%, and edible prices by 48.37%.

### 3.2. Standardized Excise Taxes

Figure 1 also presents trends in standardized excise taxes (inflation-adjusted to 2024 dollars) by product category. Standardized excise taxes also declined significantly between 2020 and 2024 (all *Ps* for linear time trends < 0.001), with a greater rate of decline than retail prices: flower taxes decreased by 53.04%, vaping taxes by 67.61%, and edible taxes by 65.07%. Over the study period, the average standardized tax was USD 32.58/ounce for flower products, USD 180.21/ounce for vaping products, and USD 0.024/milligram THC for edibles (Table 1).

**Figure 2:**
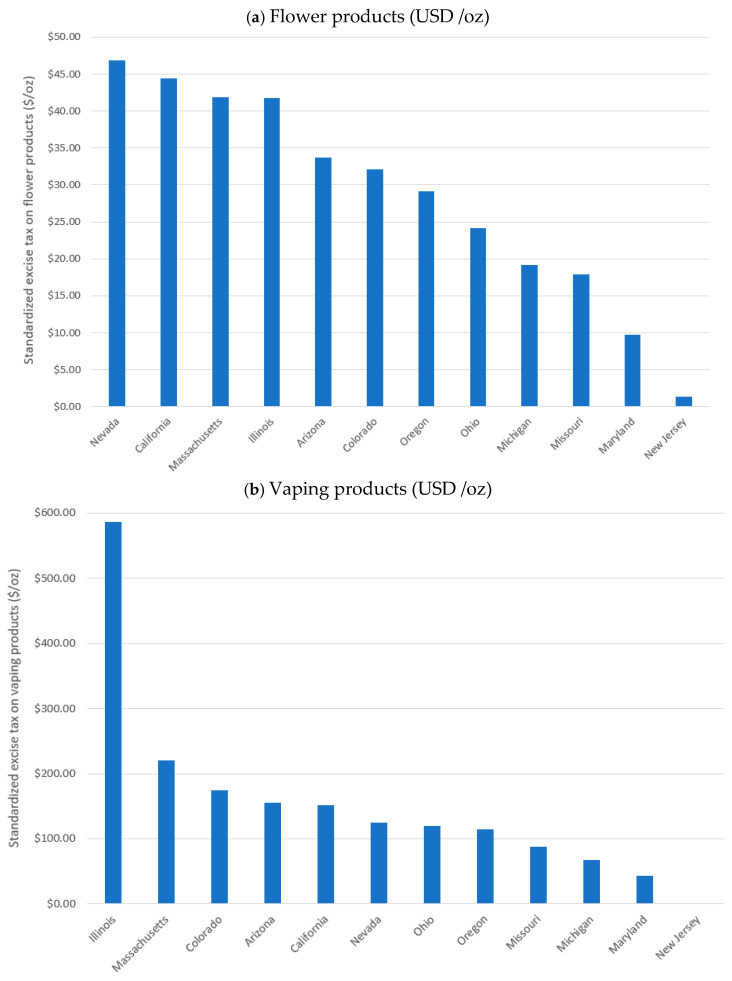

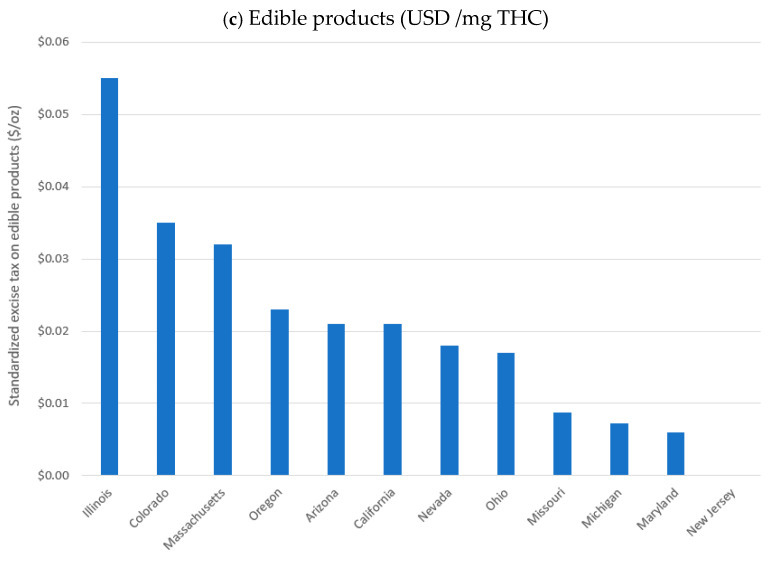
Standardized Excise Tax Magnitude by Product Category and State (inflation adjusted to 2024 dollars). (**a**): flower products, (**b**): vaping products, (**c**): edible products.

Figure 2 presents category-specific standardized excise taxes by state (inflation adjusted to 2024 dollars). For flower products, Nevada imposed the highest average excise taxes at approximately USD 46.81/ounce, while New Jersey recorded the lowest, at just USD 1.34/ounce. For vaping products, Illinois had the highest taxes, averaging USD 586.37/ounce, whereas New Jersey did not impose any excise taxes on vaping products during the study period. A similar pattern was observed for edible products. Illinois applied the highest average tax at USD 0.055/milligram THC, while New Jersey again imposed no excise taxes on this category.

Table S2 reports detailed standardized excise tax magnitudes in inflation-adjusted and unadjusted terms as well as alternative time-invariant tax measures by product category, state, and year-quarter.

### 3.3. Tax Incidence and Its Association with Tax Structure

We observed broadly comparable average tax incidence across product categories over the study period (Table 1). On average, tax incidence was 13.03% for flower products, indicating that roughly 13 cents of every dollar spent was attributable to excise taxes. The corresponding averages were 13.59% for vaping products and 13.09% for edibles.

**Figure 3:**
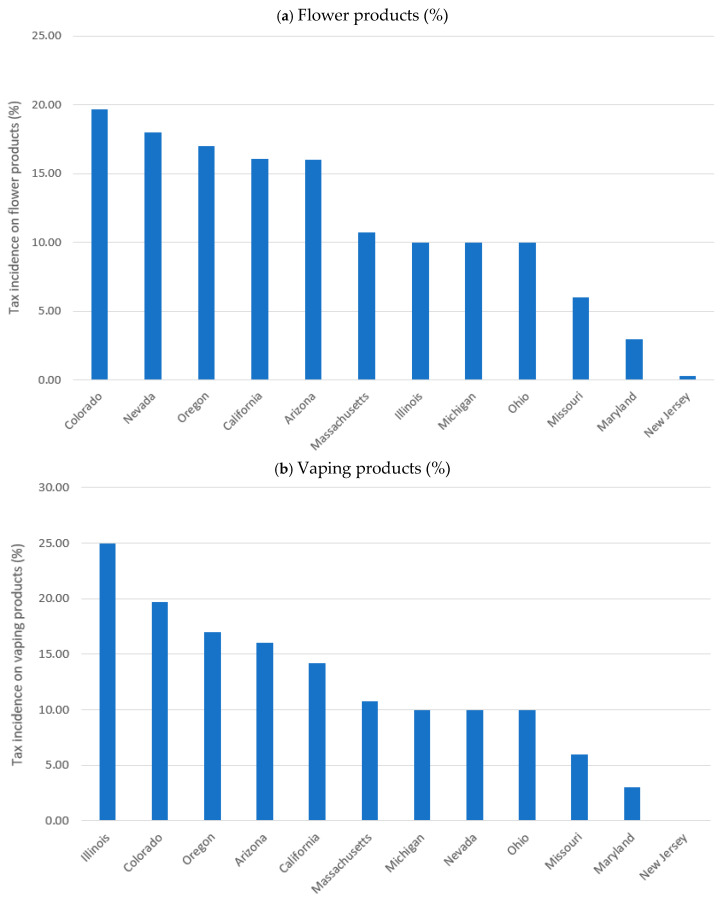

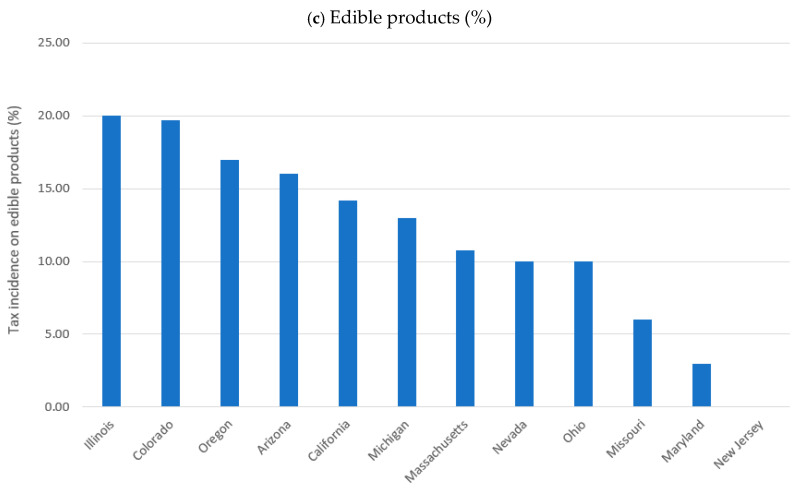
Cannabis Taxes Incidence by Product Category and State (inflation adjusted to 2024 dollars). Notes: Tax incidence (%) was computed as standardized excise taxes divided by retail prices. (**a**): flower products, (**b**): vaping products, (**c**): edible products.

Figure 3 illustrates variation in average tax incidence by product category and state. For flower products, Colorado recorded the highest incidence at 19.71%, and New Jersey reported the lowest at 0.28%. For both vaping products and edibles, Illinois showed the highest tax incidence at 25% and 20%, respectively, while New Jersey recorded 0%.

Table 2 presents the association between tax incidence and standardized excise tax magnitudes and indicators of tax bases, stratified by product category. Standardized excise tax magnitudes were significantly associated with tax incidence for both vaping products and edibles. Specifically, a USD 1 increase in the excise tax per ounce was associated with a 0.022-percentage point increase in tax incidence (95% CI: 0.014, 0.030) for vaping products. Similarly, a USD 0.01 increase in the excise tax rate per milligram of THC corresponded to a 2.26-percentage point increase in tax incidence (95% CI: 1.09, 3.43) for edibles. Tax base was associated with tax incidence across all three product categories. For flower products, mixed (combining price-based tax base with other tax bases) and price-based tax bases were linked to 14.00- and 10.54-percentage point (95% CI: 1.34, 26.66; 95% CI: 1.41, 19.67) higher tax incidence compared to a weight-based tax. For vaping products, both mixed and price-based tax bases were associated with significantly higher tax incidence: 8.45 percentage points (95% CI: 5.66, 11.24) and 10.98 percentage points (95% CI: 6.67, 15.30), respectively, relative to weight-based tax. For edibles, mixed and price-based tax bases were also significantly associated with higher tax incidence, with coefficients of 7.80 (95% CI: 4.47, 11.14) and 8.98 (95% CI: 4.54, 13.42), respectively.

### 3.4. Validity of Taxes as an Instrumental Variable for Prices

Table 3 reports the regression results using our standardized excise taxes as a predictor for retail prices. An association between taxes and prices is required for taxes serving as a valid instrumental variable for prices. Across all product categories, the category-specific tax measures developed in this study were significantly associated with retail prices, with *F*-statistics considerably exceeding the conventional threshold of 10 for an acceptable instrumental variable (15.84 for flower products, 697.90 for vaping products, and 333.65 for edibles).

Table S3 reports the regression results using our standardized excise taxes on flower products as a predictor for wholesale prices of flower products in Park et al. 2024 [10]. Our standardized excise taxes again had *F*-statistics (50.67) exceeding the conventional threshold of 10 for an acceptable instrumental variable.

### 3.5. Comparison Between Park et al. Tax Measures and Our Tax Measures

Table S4 compares the magnitudes of standardized excise taxes calculated in our study with those reported by Park et al. 2024 for the 2020–2023 period [10]. The comparison was limited to flower products, as Park et al. 2024’s [10] measures were derived for flower only. With a few exceptions, the excise taxes calculated in the present study were higher than those reported by Park et al. 2024 [10].

Table 3 also reports the regression results using tax measures on flower products from Park et al. 2024 as a predictor for retail prices for the three product categories [10]. Although Park’s tax measures were specific to flower, flower-based tax measures might still predict vaping or edible product prices if prices across product categories moved together. However, unlike our measures, Park’s tax measures showed no significant association with retail prices of flower, vaping, or edible products, and the corresponding *F*-statistics were all below the conventional threshold of 10. Park et al. 2024’s [10] flower-specific tax measures appeared to serve as an acceptable instrument for the wholesale prices of flower products in Park et al. 2024 [10], with *F*-statistics of 41.61 (Table S3).

## 4. Discussions

Building on the framework established by Park et al. 2024 [10], this study incorporated variation in prices and tax structures across cannabis product categories and utilized retail scanner data to construct category-specific standardized tax metrics for flower, vaping, and edible products, which together accounted for nearly 90% of the U.S. legal cannabis market in 2024. The analysis spanned 12 of the 22 states with legalized recreational cannabis markets between 2020 and 2024, providing a foundation for cross-state and cross-category comparisons.

Several findings are noteworthy. This study documented a consistent downward trend in excise taxes across all cannabis product categories, largely driven by the proportional nature of price-based excise taxes. The sustained reduction in both prices and taxes carries important public health implications. Consistent with economic demand theory, lower prices or taxes reduce the financial barrier to consumption, thereby increasing demand. Evidence from tobacco research further suggested that this price responsiveness was particularly pronounced among price-sensitive populations, such as adolescents and heavy users [25]. Continued declines in cannabis prices and taxes may therefore undermine states’ ability to generate sustainable tax revenue and weaken tax-based strategies aimed at curbing excessive or early cannabis use. Policy alternatives such as price floors could help stabilize retail prices, while shifting from price-based to THC- or weight-based tax structures may further reduce the extent to which excise taxes decline alongside retail prices.

We found substantial variation in cannabis excise tax incidence across states, underscoring major inconsistencies in how state governments design and implement cannabis tax policies. Such variation creates highly uneven retail environments, which may contribute to inequitable outcomes: residents in low-tax states have easier access to cheaper products, potentially elevating the risk of cannabis-related harms. In addition, large cross-state tax disparities may encourage cross-border shopping or illicit trade in cannabis products, replicating patterns long observed in tobacco markets [26].

The estimated tax incidence was 13.03% for flower, 13.59% for vaping, and 13.09% for edible products. These levels are substantially lower than those observed for tobacco products. For example, tax incidence has been estimated at approximately 28% for cigarettes, 26–32% for e-cigarettes, and 19.9–26.7% for smokeless tobacco [27,28]. The comparatively low tax incidence on cannabis suggested greater affordability for consumers, which may in turn limit the effectiveness of excise taxation as a policy tool to curb consumption and reduce cannabis-related health risks.

This study also demonstrated the potential of category-specific cannabis excise taxes as instruments for retail prices in state-level analysis, with robust associations observed across all product categories. Unlike our measures, which performed well for both wholesale and retail prices, the flower-specific tax measures developed by Park et al. 2024 with wholesale prices only predicted wholesale prices of flower products [10]. Several factors may explain these differences. First, Park et al. 2024’s [10] tax measures relied heavily on wholesale price data, which may not accurately capture actual retail prices. Second, their flower-specific measures did not account for the distinct tax structures applied to other product categories, thereby limiting their validity for category-specific analyses. From a methodological perspective, our findings underscore the importance of using category-specific, market-reflective retail pricing and tax data when applying excise taxes as instruments in causal inference models of price-related analyses.

This study had several limitations, most of which stemmed from data constraints. First, the analysis was restricted to 12 of the 22 states that had legalized recreational cannabis retail during the study period and were tracked by BDSA. Second, the BDSA retail price data relied on projections of overall market activity from a sample of dispensaries in each state. The extent to which this sample represented the full universe of dispensaries is uncertain due to the proprietary nature of the data. While direct validation was not possible, the BDSA sample included dispensaries of different sizes and types across urban, suburban, and rural areas, covering approximately 40–50% of licensed dispensaries in each state. Comparable projection-based methods have been employed in prior studies [16,29]. Third, we extrapolated wholesale and cultivation prices from observed retail prices using a uniform markup rate. Although this approach followed the precedent set by Park et al. 2024 [10], it may not fully capture variation in wholesale–retail markups across product categories and states. Fourth, the analysis did not account for local variations in excise tax policies within states. Fifth, CBD-only edible products were excluded because THC information was required for the analysis. This limitation is minor, however, as CBD-only edibles represented a relatively small share of the edible market [30]. Finally, given the evolving nature of cannabis tax policies across time and jurisdictions, the findings may not be generalizable to other periods or geographic contexts.

## 5. Conclusions

In U.S. states with recreational cannabis markets, substantial heterogeneity exists in excise tax structures across both product categories and states. This study showed that standardized taxes and tax incidences varied considerably across states. It demonstrated that category-specific standardized measures of cannabis excise taxes derived from retail scanner data may provide a valuable tool for enhancing cross-state comparisons within specific product categories. Such measures may also enable future research about how category-specific taxes and prices shape consumer behavior and, ultimately, public health outcomes.

## Figures and Tables

**Table 1:** Descriptive Statistics of Cannabis Product Prices, Taxes, and Tax Incidence by Product Category (inflation adjusted to 2024 dollars).

Variables	State-Quarter N	Mean	SD	Median	Min	Max	IQR
	Panel A: Cannabis flower products (USD/oz)						
Retail price (USD /oz)	178	279.57	115.74	264.97	131.16	582.84	144.54
Standardized excise tax (USD /oz)	178	32.58	14.78	31.42	1.18	60.99	18.86
Tax incidence (%)	178	13.034	5.48	15.00	0.22	19.71	7.20
	Panel B: Cannabis vaping products (USD/oz)						
Retail price (USD /oz)	178	1344.025	746.99	1134.66	315.87	3496.18	768.48
Standardized excise tax (USD /oz)	178	180.21	171.86	131.20	0	874.04	109.60
Tax incidence (%)	178	13.59	6.51	13.71	0	25.00	7.00
	Panel C: Cannabis edible products (USD/mg THC)						
Retail price (USD /mg THC)	174	0.18	0.082	0.18	0.032	0.44	0.10
Standardized excise tax (USD /mg THC)	174	0.024	0.016	0.021	0	0.074	0.020
Tax incidence (%)	174	13.09	5.71	13.71	0	20.00	7.00

SD: Standard deviation. IQR: Interquartile range. Notes: Data were aggregated at the state-year-quarter level from Q1 2020 through Q4 2024 in 12 states with recreational cannabis commercialization. Tax incidence (%) was computed as standardized excise taxes divided by retail prices.

**Table 2:** Association of Tax Incidence with Standardized Excise Taxes and Tax Bases, by Product Category.

Variables	Flower Products	Vaping Products	Edible Products
Standardized excise tax	0.070 (−0.23, 0.37)	0.022 *** (0.014, 0.030)	226.10 *** (109.45, 342.68)
Weight base (reference)	---	---	---
Mixed base	14.00 * (1.34, 26.66)	8.45 *** (5.66, 11.24)	7.80 *** (4.47, 11.14)
Price base	10.54 * (1.41, 19.67)	10.98 ** (6.67, 15.30)	8.98 *** (4.54, 13.42)
Number of states	12	12	12
Number of state-quarter observations	178	178	174
R-squared	0.50	0.63	0.67

Notes: *** *p* < 0.001, ** *p* < 0.01, * *p* < 0.05. All regressions controlled for year-quarter fixed effects. Standard errors were clustered at the state level.

**Table 3:** Association between Standardized Excise Taxes and Category-Specific Retail Prices: Comparison between Tax Measurements Developed by Park et al. 2024 [10] and This Study: State-Quarter Level Regressions.

Variables	Category-Specific Standardized Excise Taxes in This Study			Standardized Excise Taxes on Flower Products in Park et al. 2024 [10]		
	Flower Products	Vaping Products	Edible Products	Flower Products	Vaping Products	Edible Products
Standardized excise taxes	0.71 *** (0.35, 1.067)	0.97 *** (0.89, 1.039)	0.94 *** (0.83, 1.040)	0.30 ** (0.10, 0.52)	0.28 ** (0.074, 0.49)	0.12 (−0.14, 0.37)
Number of states	10	9	9	10	10	10
Number of state-quarter observations	128	121	117	128	128	124
R-squared	0.989	0.999	0.999	0.975	0.983	0.969
*F*-statistics	15.84	697.90	333.65	7.47	7.45	0.83

Notes: *** *p* < 0.001, ** *p* < 0.01. Cannabis prices and taxes were log-transformed. Standard errors were clustered at the state level. The comparison was restricted to overlapping states and years in the two studies (10 states in 2020–2023). All analyses also controlled for state-level sociodemographic variables, state fixed effects, and year-quarter fixed effects.

## Data Availability

Restrictions apply to the availability of the retailer scanner data. The retailer scanner data were obtained from BDSA and are available from BDSA upon request, pending a data use agreement and a data subscription. Other data are contained within the article or Supplementary Materials.

## Supplementary Materials

The following supporting information can be downloaded at: https://www.mdpi.com/article/10.3390/ijerph23010114/s1, Technical Note S1: An Example of Calculating Effective Price-based Tax Rate at the Retail Level in States Exempting and Not Exempting General Sales Taxes; Figure S1: Market Share by Product Category, 2020–2024 (%); Table S1: Overview of State-Level Cannabis Tax Policy; Table S2: Standardized Tax Measures; Table S3: Association between Standardized Excise Taxes and Flower Wholesale Prices: Comparison between Tax Measurements Developed by Park et al. 2024 [10] and This Study: State-Quarter Level Regressions; Table S4. Comparison of Standardized Excise Taxes on Flower Products Calculated from Park et al. 2024 [10] and This Study. References [31,32,33,34,35,36,37,38,39,40,41,42,43,44,45,46,47,48,49,50,51,52] are cited in the supplementary materials.

## References

[1] Walker M., Carpino M., Lightfoot D., Rossi E., Tang M., Mann R., Saarela O., Cusimano M.D. (2023). The effect of recreational cannabis legalization and commercialization on substance use, mental health, and injury: A systematic review. Public Health.

[2] Aletraris L., Graves B.D., Ndung’u J.J. (2023). Assessing the Impact of Recreational Cannabis Legalization on Cannabis Use Disorder and Admissions to Treatment in the United States. Curr. Addict. Rep..

[3] Hall W., Lynskey M. (2020). Assessing the public health impacts of legalizing recreational cannabis use: The US experience. World Psychiatry.

[4] Chaloupka F.J., Powell L.M., Warner K.E. (2019). The Use of Excise Taxes to Reduce Tobacco, Alcohol, and Sugary Beverage Consumption. Annu. Rev. Public Health.

[5] Elder R.W., Lawrence B., Ferguson A., Naimi T.S., Brewer R.D., Chattopadhyay S.K., Toomey T.L., Fielding J.E., Task Force on Community Preventive Services (2010). The effectiveness of tax policy interventions for reducing excessive alcohol consumption and related harms. Am. J. Prev. Med..

[6] Siegel M. (2002). The effectiveness of state-level tobacco control interventions: A review of program implementation and behavioral outcomes. Annu. Rev. Public Health.

[7] Hoffer A., Macumber-Rosin J. (2025). Recreational Marijuana Taxes by State. https://taxfoundation.org/data/all/state/recreational-marijuana-taxes/.

[8] Balwicki L., Stoklosa M., Balwicka-Szczyrba M., Drope J. (2020). Legal Steps to Secure the Tobacco Supply Chain: A Case Study of Poland. Int. J. Env. Res. Public Health.

[9] Wright A., Smith K.E., Hellowell M. (2017). Policy lessons from health taxes: A systematic review of empirical studies. BMC Public Health.

[10] Park H., Yoon D.W., Yang Q., He Y., Han B., Shi Y., Shang C. (2024). Recreational cannabis excise taxation in the USA: Constructing a comparable tax measure for empirical analysis. Int. J. Drug Policy.

[11] Grand View Research (2023). Legal Marijuana Market Size, Share & Trends Analysis Report by Application (Medical, Adult Use), by Product Type (Flower, Oils and Tinctures), by Region, and Segment Forecasts, 2024–2030.

[12] Grossman E.R., Deeds B., Blanco C. (2024). Tax Policy-An Understudied Approach to Reducing Cannabis Use. JAMA Psychiatry.

[13] Jawad M., Lee J.T., Glantz S., Millett C. (2018). Price elasticity of demand of non-cigarette tobacco products: A systematic review and meta-analysis. Tob. Control.

[14] Han B., Park H., He Y., Shang C., Shi Y. (2025). Estimating the Price Elasticity of Cannabis Use Among U.S. Adults: Evidence from States with Recreational Cannabis Commercialization. Cannabis Cannabinoid Res..

[15] Han B., Park H., He Y., Shang C., Shi Y. (2025). Estimating Price Elasticity of Cannabis Use Among U.S. Adolescents: Evidence From States With Recreational Cannabis Commercialization. J. Adolesc. Health.

[16] Pechmann C., Calder D., Timberlake D., Rhee J., Padon A., Silver L. (2024). Young adult retail purchases of cannabis, product category preferences and sales trends in California 2018-21: Differences compared with older adults. Addiction.

[17] Smart R., Caulkins J.P., Kilmer B., Davenport S., Midgette G. (2017). Variation in cannabis potency and prices in a newly legal market: Evidence from 30 million cannabis sales in Washington state. Addiction.

[18] Han B., Shi Y. (2025). Multilevel determinants of cannabis prices in legal markets: Evidence from products sold in nearly 4000 cannabis dispensaries in the United States. Int. J. Drug Policy.

[19] Klitzner M. Improving the Measurement of State Alcohol Taxes. https://alcoholpolicy.niaaa.nih.gov/file-page/improving-the-measurement-of-state-alcohol-taxes/79.

[20] Mahamad S., Wadsworth E., Rynard V., Goodman S., Hammond D. (2020). Availability, retail price and potency of legal and illegal cannabis in Canada after recreational cannabis legalisation. Drug Alcohol. Rev..

[21] Lamy F.R., Daniulaityte R., Zatreh M., Nahhas R.W., Sheth A., Martins S.S., Boyer E.W., Carlson R.G. (2018). “You got to love rosin: Solventless dabs, pure, clean, natural medicine.” Exploring Twitter data on emerging trends in Rosin Tech marijuana concentrates. Drug Alcohol Depend..

[22] Cinnamon Bidwell L., YorkWilliams S.L., Mueller R.L., Bryan A.D., Hutchison K.E. (2018). Exploring cannabis concentrates on the legal market: User profiles, product strength, and health-related outcomes. Addict. Behav. Rep..

[23] WHO (World Health Organization) (2013). Guidelines for Implementation of Article 6 of the WHO FCTC: Price and Tax Measures to Reduce Demand for Tobacco.

[24] Staiger D.O., Stock J.H. (1994). Instrumental variables regression with weak instruments. Econometrica.

[25] Liang L., Chaloupka F.J. (2002). Differential effects of cigarette price on youth smoking intensity. Nicotine Tob. Res..

[26] Majmundar M., Reuter P. (2015). Understanding the US Illicit Tobacco Market: Characteristics, Policy Context, and Lessons from International Experiences.

[27] Shang C., Ma S., Lindblom E.N. (2023). Tax incidence of electronic nicotine delivery systems (ENDS) in the USA. Tob. Control.

[28] He Y., Yang Q., Shang C. (2024). The Tax Incidence and Tax Pass-Through of Smokeless Tobacco in the US. Int. J. Env. Res. Public Health.

[29] Cotti C., Courtemanche C., Maclean J.C., Nesson E., Pesko M.F., Tefft N.W. (2022). The effects of e-cigarette taxes on e-cigarette prices and tobacco product sales: Evidence from retail panel data. J. Health Econ..

[30] Han B., Shi Y. (2025). A content analysis of cannabis edible product characteristics, packaging features, and online promotions. Prev. Med..

[31] Alaska Department of Revenue Marijuana Tax. https://tax.alaska.gov/programs/programs/index.aspx?60000.

[32] Arizona Department of Revenue Marijuana Tax Collection. https://azdor.gov/reports-statistics-and-legal-research/marijuana-tax-collection.

[33] California Department of Tax and Fee Administration Tax Guide for Cannabis Business. https://cdtfa.ca.gov/industry/cannabis/.

[34] Colorado Department of Revenue Marijuana Tax Reports. https://cdor.colorado.gov/data-and-reports/marijuana-data/marijuana-tax-reports.

[35] Connecticut State Department of Revenue Services Cannabis Tax Information. https://portal.ct.gov/DRS/Taxes/Cannabis/Cannabis-Tax.

[36] Illinois Department of Revenue Cannabis Tax. https://tax.illinois.gov/research/taxinformation/other/cannabis-taxes.html.

[37] Maine Department of Adminisrtative and Financial Services Title 36: Taxation. https://www.maine.gov/dafs/ocp/adult-use/rules-statutes/title-36.

[38] Maryland Cannabis Administration Adult-Use Cannabis FAQs. https://cannabis.maryland.gov/pages/cannabisfaq.aspx.

[39] Massachusetts Cannabis Control Commission Taxes and Fees. https://masscannabiscontrol.com/taxes-and-fees/.

[40] Michigan Revenue Adminisrtative Bulletins Taxation of Adult-Use (Recreational) Marihuana under the Michigan Regulation and Taxation of Marihuana Act. https://www.michigan.gov/taxes/rep-legal/rab/rabhtml/2020/revenue-administrative-bulletin-2020-17#_ftn9.

[41] Minnesota Department of Revenue Cannabis Tax. https://www.revenue.state.mn.us/cannabis-tax.

[42] Missouri Department of Revenue Marijuana. https://dor.mo.gov/taxation/business/marijuana.html#:~:text=Article%20XIV%20of%20the%20Missouri%20Constitution%20applies%20a%206%25%20tax,dispensary%20facilities%20within%20the%20state..

[43] Montanna Department of Revenue Cannabis Tax. https://revenue.mt.gov/taxes/miscellaneous-taxes-and-fees/cannabis/.

[44] Nevada Department of Taxation Cannabis Tax. https://tax.nv.gov/tax-types/cannabis-tax/.

[45] New Jersey Treasury Recreational Cannabis. https://www.nj.gov/treasury/taxation/cannabis/recreational/index.shtml.

[46] New Mexico Taxation and Revenue Cannabis Excise Tax. https://www.tax.newmexico.gov/businesses/cannabis-excise-tax/.

[47] New York State Department of Taxation and Finance Adult-Use Cannabis Product Tax. https://www.tax.ny.gov/bus/auc/#:~:text=No.,who%20sells%20adult%2Duse%20cannabis.

[48] Ohio Department of Taxation Adult Use Cannabis Tax. https://tax.ohio.gov/business/adult-use-cannabis-tax.

[49] Oregon Department of Revenue Statistics from Oregon Marijuana Tax Returns. https://www.oregon.gov/dor/programs/gov-research/Documents/marijuana-tax-report_2016.pdf.

[50] Rhode Island Department of Revenue Adult Use Cannabis Tax. https://tax.ri.gov/tax-sections/sales-excise-taxes/adult-use-cannabis-tax.

[51] Vermont Department of Taxation Cannabis Excise Tax. https://tax.vermont.gov/business/cannabis-excise-tax.

[52] Washington State Department of Revenue Taxes Due on Cannabis. https://dor.wa.gov/taxes-rates/taxes-due-cannabis.

