# Supplementary material for: Standardizing Recreational Cannabis Excise Tax Rates in the United States: New Retail Price-Based Measurements by Product Category
Source: Int J Environ Res Public Health. 2026 Jan 16;23(1):114. doi: 10.3390/ijerph23010114 (PMC12840896; doi:10.3390/ijerph23010114)
Supplement: Supplementary file 1 [file ijerph-23-00114-s001.zip › Supplementary 01-14-2025.pdf]

Supplementary Materials

Technical Note S1. An Example of Calculating Effective Price-based Tax Rate at the Retail Level in States Exempting and Not Exempting General Sales Taxes

State	State-level general sales tax rate	Retail-level price-based excise tax rate on cannabis	Is state-level general sales tax applied to cannabis?	Adjusted retail-level price-based excise tax rate on cannabis (Actual tax burden)
A	6%	10%	Yes	10%
B	6%	10%	No	4% (10%-6%)

General state sales taxes may or may not apply to cannabis products and are typically paid by consumers at the point of sale. Cannabis-specific excise taxes at the retail level are calculated as a percentage of the retail prices and typically remitted by retailers to the state. In states where cannabis is exempt from the general sales taxes, the taxes are not part of the total tax burden, whereas in states where cannabis is not exempt, they are included. To ensure comparability of effective tax burdens across states, we adjusted price-based excise taxes to account for whether general sales taxes apply to cannabis.

For example, consider two states that both levy retail-level price-based cannabis excise taxes with a tax rate of 10%. In State A, cannabis is also subject to 6% general sales taxes, while in State B, cannabis is exempt from the sales taxes. Although the statutory excise tax rate is the same in both states, the overall tax burden differs. To make these tax burdens comparable, we adjust the effective excise tax rate in the tax-exempt State B by subtracting the sales taxes that would otherwise apply, yielding an adjusted rate of 4% in State B (10% minus 6%). Without this adjustment, tax burdens in the two states would appear equivalent even though State B has a lower overall tax burden. This approach follows the method used in Park et al. 2024 [10] and is consistent with practices adopted by NIAAA [19] in constructing effective excise tax rates for alcoholic beverages, for which sales tax exemptions are common.

Figure S1. Market Share by Product Category, 2020–2024 (%)

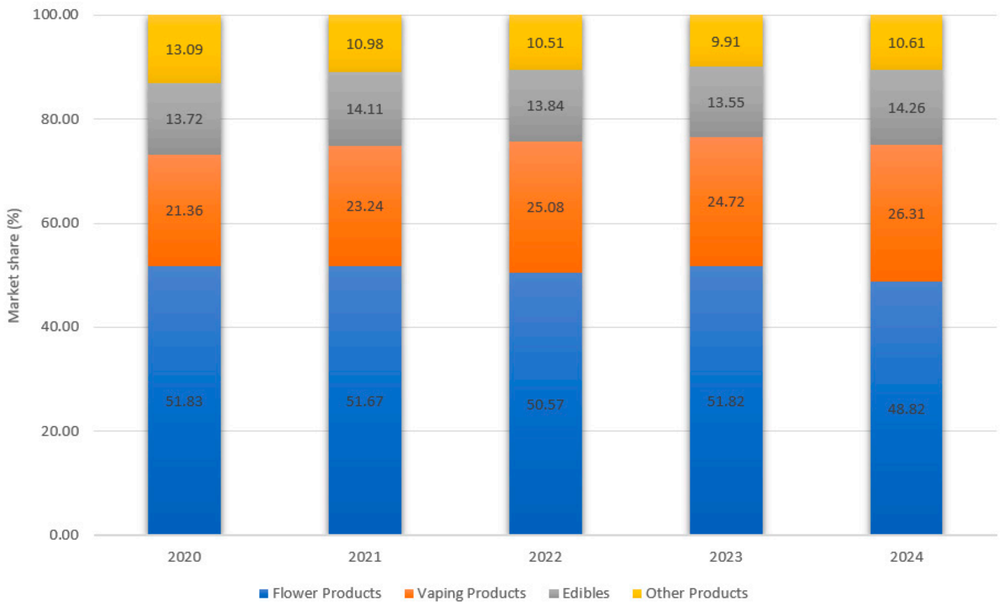

Notes: Market share for each product category was calculated as the total sales of that category divided by the combined total sales of all product categories.

Table S3. Association between Standardized Excise Taxes and Flower Wholesale Prices: Comparison between Tax Measurements Developed by Park et al. 2024 [10] and This Study: State-Quarter Level Regressions

**Variables**	**Flower-specific Standardized Excise Taxes in This Study**	**Flower-specific Standardized Excise Taxes in Park et al. 2024 [10]**
Standardized excise taxes	1.17***<br>(0.84, 1.50)	0.83***<br>(0.57, 1.10)
State-level sociodemographic controls	Yes	Yes
State fixed effects	Yes	Yes
Year-quarter fixed effects	Yes	Yes
Number of states	9	9
Number of state-quarter observations	121	121
R-squared	0.969	0.989
***F*-statistics**	50.67	41.61

Notes: *** p<0.001. Cannabis prices and taxes were log-transformed. Standard errors were clustered at the state level. The comparison was restricted to overlapping states and years in the two studies (9 states in 2020-2023).

Table S4. Comparison of Standardized Excise Taxes on Flower Products Calculated from Park et al. 2024 [10] and This Study (inflation adjusted to 2024 dollars)

State	Year	Standardized Tax (Park et al. 2024) [10] ($/oz)	Standardized Tax (This Study) ($/oz)	Difference Between Park et al. 2024 [10] and This Study ($/oz)
Arizona	2021	36.06	48.73	12.67
	2022	22.41	38.19	15.78
	2023	14.38	26.39	12.01
California	2020	32.87	55.24	22.37
	2021	31.60	50.07	18.47
	2022	18.26	45.86	27.60
	2023	13.81	38.93	25.12
Colorado	2020	27.71	36.21	8.50
	2021	29.83	33.63	3.80
	2022	20.46	34.15	13.69
	2023	18.40	30.09	11.69
Illinois	2020	60.52	56.03	-4.49
	2021	57.18	47.49	-9.69
	2022	51.18	42.95	-8.23
	2023	39.57	32.69	-6.88
Maryland	2023	7.51	10.51	3.00
Massachusetts	2020	42.40	52.63	10.23
	2021	42.04	49.34	7.30
	2022	31.08	48.54	17.46
	2023	18.48	31.92	13.44
Michigan	2021	22.89	27.25	4.36
	2022	12.72	19.18	6.46
	2023	9.20	14.86	5.66
Nevada	2020	49.26	55.72	6.46
	2021	48.79	52.77	3.98
	2022	49.19	49.11	-0.08
	2023	39.15	41.67	2.52
New Jersey	2022	1.10	1.10	0
	2023	1.52	1.52	0
Oregon	2020	25.76	30.16	4.40
	2021	25.06	28.84	3.78
	2022	20.86	30.58	9.72
	2023	18.62	27.42	8.80

Notes: The comparison was restricted to overlapping states and years in the two studies.
